# A Randomized, Double-Blind, Placebo-Controlled Trial of an Ayurvedic Herbal Formulation and Vitamin C/E on Vascular Function in Patients with Cardiovascular Disease

**DOI:** 10.3390/medicina62050972

**Published:** 2026-05-15

**Authors:** John W. Salerno, Shichen Xu, Maxwell Rainforth, Sanford I. Nidich, Robert H. Schneider

**Affiliations:** 1 Institute for Prevention Research, Vedic City, IA 52556, USA; jsalerno@miu.edu (J.W.S.);; 2 Howard University Hospital Heart Center, Washington, DC 20060, USA; 3 Center for Natural Medicine and Prevention, Maharishi International University, Fairfield, IA 52556, USA

**Keywords:** ayurveda, cardiovascular disease, endothelial function, vitamin C, vitamin E, herbal medicine

## Abstract

*Background and Objectives*: Cardiovascular disease (CVD) is the leading cause of death globally. The World Health Organization has called for investigations into traditional systems of medicine for CVD prevention. Ayurveda includes a classical herbal formulation called Maharishi Amrit Kalash (MAK) traditionally used for disease prevention, health promotion and healthy aging. The study objective was to evaluate MAK effects on biomarkers of vascular function and structure compared to vitamin C and E supplementation in a high CVD risk population. *Materials and Methods*: In this double-blind randomized controlled trial, 138 Black men and women (mean age 65 ± 7 years) with established CVD or high CVD risk were assigned to either MAK (*n* = 46), vitamin C/E (*n* = 46), or placebo (*n* = 46) for 12 months. The primary outcomes were change in brachial artery reactivity testing (BART) with flow-mediated dilation (FMD, endothelium-dependent) and nitroglycerin-mediated dilation (NMD, endothelium-independent). Other outcomes included carotid intima-media thickness (cIMT), blood pressure, and serum lipids. ANCOVA and pairwise comparisons were performed. *Results*: After 12 months of intervention, the MAK group demonstrated significant improvement in BART-NMD compared to placebo (mean change + 4.18% vs. +2.95%, *p* = 0.018) and numerical but non-significant improvement compared to the +3.32% mean change for the Vitamin C/E group (*p* = NS). There were no significant group differences for BART-FMD, cIMT, blood pressure, and lipids. Intervention compliance ranged from 70–80%. *Conclusions*: In this randomized controlled trial, 12 months of MAK supplementation improved endothelium-independent vascular smooth muscle function (BART-NMD) in Black adults at high CVD risk. The MAK group achieved a mean BART-NMD of approximately 15.6%, reaching the established threshold for normal vascular smooth muscle function. This selective improvement in smooth muscle responsiveness without changes in endothelial function, vascular structure, or conventional risk factors suggests MAK may influence specific pathways relevant to vascular aging. Larger studies with clinical outcomes are needed to further evaluate this effect on cardiovascular health in aging and high-risk populations.

## 1. Introduction

Cardiovascular disease (CVD) remains the leading cause of mortality worldwide and represents a major contributor to disability and healthcare utilization in aging populations [1]. The incidence and prevalence of CVD rise sharply with advancing age, reflecting progressive alterations in vascular structure and function, accumulation of metabolic stressors, and declining physiological reserve. These age-related changes contribute to the development of hypertension, atherosclerosis, ischemic heart disease, and heart failure that account for the largest share of the global burden of disease [1].

In the United States, CVD contributes substantially to health disparities, with Black men and women experiencing approximately 50% higher mortality than the general population [1]. These observations highlight the importance of preventive strategies that address vascular dysfunction and disease in aging and in high-risk populations.

Chronic age-associated CVD involves the interaction of multiple biological processes rather than a single dominant pathway. Preclinical and clinical literature highlight contributions from metabolic dysregulation, endothelial function, vascular smooth muscle responsiveness, and oxidative and inflammatory signaling [2,3,4]. While conventional therapies may effectively target specific components of cardiovascular risk, such as lipids, blood pressure, and thrombosis the multifactorial nature of vascular aging has prompted investigation of additional approaches that may influence multiple pathways simultaneously. Accordingly, there is increasing scientific interest in whether multi-component strategies may complement conventional single-target approaches [2,5].

Dietary supplements and nutraceuticals have been widely explored as adjunctive strategies for cardiovascular prevention [2]. Among these, isolated antioxidant vitamins, including vitamins C and E, have been evaluated in large randomized trials. However, results have been inconsistent, and contemporary scientific statements do not support routine vitamin supplementation for cardiovascular risk reduction [5,6]. These findings underscore the challenges of modifying complex age-related disease processes with single agents with isolated targets and have supported further investigation into multi-component formulations.

In parallel, utilization of traditional, complementary, and integrative medicine (TCIM) has increased globally, particularly for chronic and age-related conditions. The World Health Organization and other international health organizations have emphasized the need for rigorous scientific evaluation of traditional medical systems to clarify their potential roles in disease prevention, healthy aging, and chronic disease management [7,8]. This perspective was articulated by Seetharaman et al. in *‘* The Future of Medicine: Frontiers in Integrative Health and Medicine,’ which emphasized population aging, non-communicable disease burden, and systems-based investigation as key drivers for research in TCIM [9].

Ayurveda is a traditional system of medicine with a long-standing emphasis on prevention, functional maintenance, and aging-related health. A central therapeutic category within Ayurveda is *rasayana*, which historically refers to interventions used to support longevity, physiological function, and resistance to disease [10]. *Rasayana* formulations are typically multi-component preparations composed of diverse botanical and nutritive substances. Contemporary experimental and clinical literature suggests that such formulations may influence biological processes relevant to age-associated disease, including oxidative balance, immune regulation, and metabolic function [11,12].

Maharishi Amrit Kalash (MAK) is a standardized *rasayana* formulation consisting of two complementary preparations (MAK-4 and MAK-5) that together contain more than 50 botanicals, including *Emblica officinalis*, *Withania somnifera*, and *Centella asiatica*. Preclinical studies have demonstrated antioxidant activity, inhibition of low-density lipoprotein oxidation, and anti-atherogenic effects [12,13,14]. Early clinical investigations reported improvements in angina symptoms, oxidative biomarkers, and carotid atherosclerosis progression either alone or in combination with lifestyle modifications [15,16]. More recent systematic and scoping reviews have summarized evidence suggesting potential roles of MAK in cardiometabolic and other age-related conditions [12,17,18]. These data support further evaluation of MAK using contemporary clinical research methods.

Vascular dysfunction is a hallmark of both vascular aging and atherosclerosis. It is clearly associated with incident cardiovascular events and mortality [19,20]. Brachial artery reactivity testing (BART) provides a validated, non-invasive assessment of both endothelium-dependent flow-mediated dilation (BART-FMD) and endothelium-independent nitroglycerin-mediated dilation (BART-NMD), allowing evaluation of complementary components of vascular function relevant to cardiovascular risk [21,22].

The objective of this clinical trial was to determine whether supplementation with the traditional medicine *rasayana* formulation Maharishi Amrit Kalash improves vascular function compared with both placebo and a reductionist antioxidant strategy (vitamin C/E) in adults with established or high risk for CVD. We hypothesized that MAK would favorably influence vascular reactivity, reflecting the potential of a multi-component traditional formulation to modulate biological processes relevant to vascular aging and cardiovascular risk.

## 2. Materials and Methods

### 2.1. Design Overview

This was a randomized, double-blind, placebo-controlled three-arm clinical trial. Baseline and post-testing were conducted before and after 12 months of intervention in patients at high risk of CVD. The clinical site was Howard University Medical Center (HUMC), Washington DC for participant recruiting, program implementation and data collection. The core laboratory for ultrasound methods and analyses was Cedars Sinai Medical Center (CSMC), Los Angeles, CA. The administrative and data coordinating center (ADCC) was the Institute for Natural Medicine and Prevention, Maharishi International University (MIU), Fairfield, IA. All participants continued with their usual medical care. The study protocol was approved by the institutional review boards of HUMC and MIU. Informed consent form was given in written form by all participants. The investigations were conducted in accordance with the principles outlined in the Declaration of Helsinki (1975, revised in 2013).

This clinical trial enrolled participants from October 2002 through June 2004, with post-testing completed in July 2005. The trial began and completed enrollment before the International Committee of Medical Journal Editors (ICMJE) trial registration policy took effect, which applies to trials beginning enrollment on or after July 2005 [23]. The trial was registered retrospectively with ClinicalTrials.gov (NCT#07206901) in September 2025 to enhance transparency and accessibility of trial information. Data were securely archived and subsequently retrieved for analysis completed in December 2025. The interval between trial completion and final data analysis was due to intermittent funding and resource constraints. The trial design, conduct, and reporting reflect the CONSORT 2025 statement for randomized controlled trials and is consistent with the elaborated CONSORT statement for reporting randomized controlled trials of herbal interventions [24,25].

### 2.2. Study Participants

Inclusion criteria were male or female, self-identified Black or African American, high risk for CVD defined as either established coronary heart disease (history of myocardial infarction, coronary revascularization, or angiographic stenosis > 50%) or at least two major CVD risk factors (hypertension, diabetes, hyperlipidemia, smoking, or family history of premature CVD). Exclusion criteria included decompensated heart failure, chronic kidney or liver disease, malignancy, or contraindication to nitroglycerin.

### 2.3. Randomization, Blinding and Allocation Concealment

Participants were randomized in 1:1:1 proportion to three intervention groups: MAK, vitamin C/E, or placebo. Randomization was computer-generated, with allocation concealed in coded bottles prepared by the study pharmacy. Participants, clinicians, and outcome assessors were blinded to group assignments. The project manager at the HUMC clinical site received the random assignments (into groups 1, 2 or 3) from the biostatistician and notified the participants by phone of the time and location of their first clinic meeting to pick up their two-month supply of number coded dietary supplements.

### 2.4. Interventions

The MAK formulations used in this study were from standardized commercial products manufactured by Maharishi Ayurveda Products International (MAPI) in Noida, India. They were produced under ISO-9001 [26] certified conditions with internationally established quality control procedures to ensure batch-to-batch consistency. Although detailed phytochemical marker analyses were not available for the specific batches used in this trial, the manufacturing process included standardized sourcing, processing, and quality assurance protocols.

The MAK herbal intervention consisted of two tablets twice daily of MAK-4 and MAK-5. This is a two-part standardized formulation of 50 botanicals that was produced under quality-controlled manufacturing. MAK-4 consisted of Indian gallnut, Indian gooseberry, dried catkins, Indian pennywort, honey, nutgrass, white sandalwood, butterfly pea, shoeflower, aloewood, licorice, cardamom, cinnamon, Indian cyperus, and turmeric. MAK-5 consisted of *Gymnema aurantiacum*, *Hypoxis orchiodes*, *Tinospora cordifolia*, *Sphaeranthus indicus*, butterfly pea, licorice, *Vanda spatulatum*, *Lettsomia nervosa*, and Indian wild pepper [17]. Each batch of herbs was tested for microbial and heavy metal contamination.

The vitamin C/E arm received 1000 mg vitamin C and 400 IU vitamin E daily (Blistech Pharmaceuticals, Fairfield, NJ, USA). The MAK and vitamin C/E tablets were matched for size, shape, and appearance. Doses of vitamin C and E were similar to the doses used in prior large-scale CVD prevention trials [6]. Placebo tablets were also matched in size, shape, and appearance to MAK and Vitamin C/E.

All interventions were dispensed in identical bottles. To maintain blinding and an equivalent pill burden across the three study arms, participants in all groups received two bottles at each study visit. The MAK and vitamin C/E interventions each consisted of two tablets of differing size, The placebo was designed to mirror this structure, with two placebo tablets of corresponding sizes. All tablets were matched in appearance (size, shape, and color coating) to their respective active comparators. A single placebo formulation was used and matched in size, shape, and color to the active tablets in both intervention arms, with two placebo tablets of corresponding sizes provided to mirror the dosing structure. The storage and distribution center for all dietary supplements was Blistech Pharmaceuticals, Fairfield, NJ, USA. Participants, study staff, clinicians, and outcome assessors were blinded to treatment allocation throughout the study.

### 2.5. Outcomes Assessment

The co-primary outcomes were changes in brachial artery reactivity testing (BART) using flow-medicated dilation, FMD and nitroglycerin-mediated dilation, NMD. BART-FMD and BART-NMD were expressed as the percentage change in brachial artery dilation. Other outcomes included carotid intima-media thickness (cIMT), office blood pressure and fasting lipids (total cholesterol, LDL, HDL, triglycerides).

BART was performed by certified sonographers using a standardized ultrasound protocol as previously described [22]. Endothelium-dependent vasodilation, BART-FMD was measured after 5 min of forearm cuff occlusion and release. Endothelium-independent vasodilation, BART-NMD was assessed 10 min later, after sublingual administration of 0.4 mg nitroglycerin. Image analysis was centralized and blinded, with inter-observer variability < 5%. Carotid IMT methods have been previously described [26]. Compliance to the interventions was assessed by pill counts and participant diaries at each two-month study visit.

Statistical analyses utilized the modified intent-to-treat principle with all data collected used in the analyses regardless of compliance with the interventions. Sample size was determined by statistical power analysis for BART at 12 months. Treatment effects and variability were based on prior studies of the effects of dietary and nonfood-derived antioxidant supplements [27]. We assumed analysis of posttest outcomes covaried for baseline with two-tailed tests at the 0.05 level, 80% statistical power, and 20% attrition over 12 months. Thus, it was estimated that 46 subjects would be required in each of the three study arms for a total N = 138 to reliably detect changes in BART. Between-group differences in 12-month changes were tested using one-way analysis of covariance (ANCOVA), adjusting for baseline values. Prespecified comparisons were MAK vs placebo, vitamin C/E vs placebo, and MAK vs vitamin C/E using Bonferroni-corrected pairwise analyses. Significance was set at *p* < 0.05 two-sided.

Participants who experienced adverse clinical events during the study were reported to the HUMC IRB within a 24 h period by the investigators to determine whether the event was related to study participation.

## 3. Results

**Figure 1:**
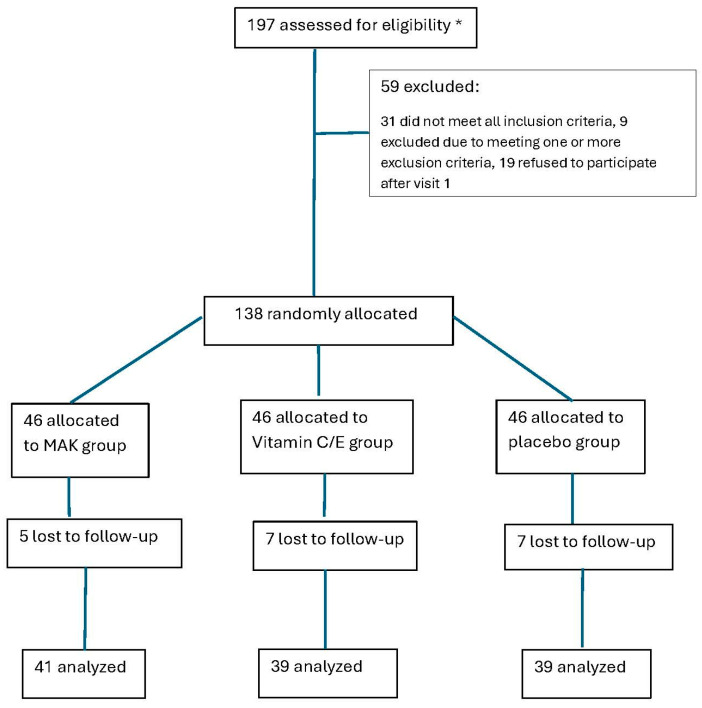
Patient Flow Diagram. * = estimated.

The participant flow diagram from eligibility through to final analysis is presented in Figure 1.

Baseline characteristics were balanced across the three groups (Table 1). The mean age was 65 ± 6.7 (range 55–80) years, 64% were women, 71% had hypertension, and 28% had diabetes. Mean BMI was 29 kg/m^2^, and average systolic blood pressure was 136 mmHg. Mean LDL cholesterol was 121 mg/dL. Baseline brachial artery dilation responses averaged 7.8% for BART-FMD and 11.4% for BART-NMD.

**Figure 2:**
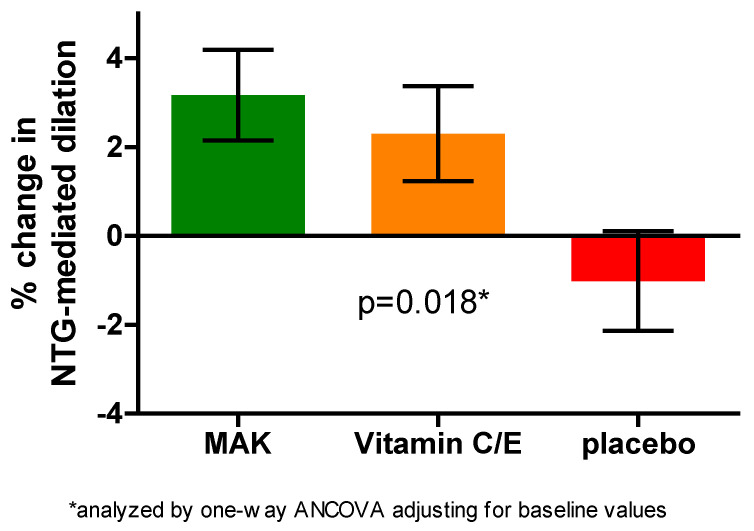
**Change** **in BART-NMD after 12 months of intervention.**

For the primary outcome of BART-NMD after 12 months, ANCOVA showed a significant difference among the three groups (*p* = 0.018). See Table 2. In the prespecified pairwise comparison, the MAK group significantly improved on % change for the BART-NMD compared to placebo (*p* < 0.05) (Table 2 and Figure 2). The vitamin C/E group did not show a significant change compared to placebo or MAK for the BART-NMD. MAK and vitamin C/E groups were not different from each other or placebo on BART-FMD changes.

Other outcomes shown in Table 2 including carotid IMT did not differ between groups. Blood pressure decreased modestly across all groups without between-group differences. Lipid levels remained stable, with no differential changes in total cholesterol, LDL, HDL or triglycerides. Compliance rates were 70% in the MAK group, 73% in the vitamin group, and 80% in the placebo group (*p* = 0.14).

There were a total of 7 serious adverse events reported across all 3 groups including 2 CVD and 5 non-CVD events. There were no fatal events. Mild gastrointestinal upset was occasionally reported in all three groups but did not lead to discontinuation.

## 4. Discussion

This randomized, double-blind, placebo-controlled trial demonstrated that 12 months of MAK supplementation improved endothelium-independent vascular function (BART-NMD) in Black adults at high risk for CVD. The enhancement in BART-NMD suggests improved vascular smooth muscle responsiveness to nitric oxide. The MAK group showed a mean improvement of +4.18% compared to +2.95% in placebo (*p* = 0.018), resulting in a final BART-NMD value of approximately 15%. This is clinically relevant because recent diagnostic criteria established from published cohorts (n = 1764) identified 15.6% as the optimal cutoff for normal vascular smooth muscle function, while values < 11.6% predict increased cardiovascular events [28]. Our study population had a baseline mean BART-NMD of 11.4%, indicating impaired smooth muscle function at entry. The MAK intervention effectively restored BART-NMD to the normal threshold, whereas placebo-treated participants remained below this threshold at approximately 14.4%.

This finding may have clinical relevance, as impaired vascular reactivity—particularly endothelium-dependent function (assessed by FMD)—has been shown to predict future cardiovascular events and all-cause mortality independent of traditional risk factors [19]. In the Cardiovascular Health Study, brachial artery flow-mediated dilation was a significant predictor of cardiovascular events over a 5-year period after adjustment for major risk factors [18]. In addition, prospective studies have demonstrated that impaired endothelium-independent vasodilation (assessed by NMD) independently predicts future cardiovascular events, even after accounting for FMD. Further, the combination of NMD and FMD improved risk prediction compared to either measure alone [29]. These findings suggest that NMD reflects complementary aspects of vascular dysfunction, including vascular smooth muscle responsiveness and structural vascular alterations associated with atherosclerosis.

These observations are further supported by prior study in this cohort demonstrating that both FMD and NMD are inversely associated with cardiovascular risk determined by the Framingham Risk Score [22]. Whereas a structural measure such as carotid IMT was less sensitive to change with MAK in the current trial. Together, these data indicate that vascular reactivity testing captures multiple dimensions of vascular biology, and that improvement in smooth muscle–mediated dilation may represent a physiologically meaningful change relevant to vascular aging and cardiovascular risk.

BART-FMD, a measure of endothelial-dependent function, did not differ between groups. This divergence indicates that MAK’s effect was at the level of vascular smooth muscle rather than the endothelium. Such a pattern aligns with preclinical evidence that oxidative stress impairs soluble guanylate cyclase activity and reduces cGMP-mediated signaling, thus reducing smooth muscle sensitivity to nitric oxide [3,30]. MAK’s diverse phytochemicals—including polyphenols, carotenoids, and flavonoids—may counteract these processes, preserving smooth muscle relaxation capacity [12].

The pattern of isolated improvement in endothelium-independent function without corresponding changes in endothelium-dependent function warrants careful interpretation. Traditionally, vascular dysfunction progresses from endothelial impairment to subsequent smooth muscle dysfunction with prolonged exposure to cardiovascular risk factors. However, recent evidence from a comprehensive coronary vasomotor dysfunction study of 1196 patients with nonobstructive coronary artery disease followed for a median of 6.3 years demonstrates that endothelium-dependent and endothelium-independent dysfunction can occur in isolation or combination, with each phenotype conferring different prognostic implications [31,32]. Specifically, endothelium-independent microvascular dysfunction independently predicted major adverse cardiovascular events and mortality across the spectrum of cardiovascular disease. The selective improvement in smooth muscle function observed with MAK may reflect the formulation’s multi-component composition, which includes diverse phytochemicals capable of modulating multiple pathways simultaneously [12].

Other outcomes of cIMT, blood pressure, and lipids were unchanged. This is consistent with prior studies of MAK and related interventions, which have shown variable or limited effects on structural vascular measures, hemodynamics, and lipid levels [17]. Nevertheless, the selective improvement in BART-NMD highlights a potential mechanism by which multi-herbal formulations could complement existing therapies.

Our results extend earlier reports on MAK. Dogra et al. demonstrated improved angina symptoms with MAK in coronary artery disease patients [15]. Lee and Sundaram found antioxidant and anti-atherogenic effects in animal and human studies [14]. Fields et al. showed reduced progression of carotid atherosclerosis with an Ayurvedic lifestyle medicine program including MAK [16]. A pooled analysis by Walton, et al. of a multimodality Ayurvedic regimen on CVD patients that included MAK supplementation along with meditation and yoga showed a significant reduction in cIMT compared to control patients in usual care [33]. Together, these data suggest a case for MAK’s vascular protective properties.

These findings are relevant for Black adult populations, who face disproportionate CVD burdens and are underrepresented in clinical research. Interventions that are natural, culturally acceptable, and well tolerated may improve engagement and adherence, addressing a critical gap in preventive cardiology. From a translational perspective, MAK represents an example of how traditional multi-herbal formulations can be standardized and rigorously tested in modern clinical settings. Compared to single ingredient vitamins, ie, vitamins C/E, multi-botanical combinations may achieve synergistic effects across multiple pathways [11].

This study had limitations. The modest sample size limited statistical power for detecting differences between MAK and vitamin C/E groups and for other outcomes including cIMT, blood pressure, or lipids. Generalizability may be limited to older Black adults in an urban location. Although the BART-NMD cutoff values were derived from Japanese cohorts, they provide a useful reference for interpreting the clinical significance of the observed changes. Adherence, though typical for a long-term trial, was moderate. Finally, the use of surrogate endpoints rather than clinical outcomes restricts conclusions about long-term benefit although previous studies suggest that BART-NMD predicts long-term CVD events [30]. The interval between study completion and final analysis may have introduced potential bias due to temporal changes, including evolving diagnostic criteria and clinical practice patterns.

## 5. Conclusions

This randomized, double-blind, placebo-controlled trial demonstrated that 12 months of supplementation with an Ayurvedic herbal *rasayana* formulation, Maharishi Amrit Kalash was associated with significant improvement in endothelium-independent vascular function in Black men and women at high risk for cardiovascular disease. Vascular smooth muscle responsiveness to nitric oxide declines with advancing age and is considered an important component of vascular aging that independently predicts cardiovascular events. The observed enhancement of nitroglycerin-mediated dilation therefore suggests a potential influence of this multi-component formulation on biological processes relevant to age-associated vascular dysfunction.

Although no effects were observed on endothelial-dependent dilation, vascular structure, or conventional risk factors, these findings demonstrate that a standardized Ayurvedic herbal formulation may selectively modulate aspects of vascular physiology linked to aging. The lack of effect from combined vitamin C/E supplementation, despite adequate dosing, highlights the limitations of reductionist antioxidant strategies and supports the potential value of multi-component traditional herbal formulations that may engage multiple pathways simultaneously.

Larger, multiethnic studies incorporating mechanistic biomarkers and clinical outcomes are suggested to clarify the role of such interventions as traditional, complementary, and integrative strategies for promoting vascular health and reducing cardiovascular disease in high-risk and aging populations.

## Figures and Tables

**Table 1:** Baseline characteristics of the study participants.

Characteristic	MAK (n = 46)	Vitamin C/E (n = 46)	Placebo (n = 46)
Age, years (mean ± SD)	65 ± 7	65 ± 6	64 ± 7
Female, %	63	64	65
BMI, kg/m^2^ (mean ± SD)	29.0 ± 4.8	29.2 ± 5.0	28.9 ± 4.5
Hypertension, %	71	70	71
Diabetes, %	28	27	29
Current smoker, %	23	21	22
LDL cholesterol, mg/dL	121 ± 34	119 ± 32	122 ± 33
Systolic BP, mmHg	136 ± 12	135 ± 13	136 ± 12
Diastolic BP, mmHg	82 ± 8	81 ± 9	82 ± 8
BART-FMD, % change	7.8 ± 3.2	7.9 ± 3.3	7.7 ± 3.1
BART-NMD, % change	11.4 ± 3.5	11.2 ± 3.6	11.5 ± 3.4

**Table 2:** Change in outcomes after 12 months of intervention.

Outcome	MAK (n = 41)	Vitamin C/E (n = 39)	Placebo (n = 39)	Difference Between Groups (*p*-Value by ANCOVA)
	mean ± SE	mean ± SE	mean ± SE	
BART-FMD (% change)	+3.95 ± 0.4 6.47	+3.72 ± 0.5 5.59	+3.60 ± 0.5 6.64	0.42
BART-NMD (% change)	+4.18 ± 0.5 4.17	+3.32 ± 0.6 4.13	+2.95 ± 0.5 4.27	0.018
Carotid IMT (mm)	+0.005 ± 0.01 0.857	−0.034 ± 0.01 0.864	−0.011 ± 0.013 0.921	0.077
Systolic BP (mm Hg)	+5.36 ± 2.96 133.5	−1.82 ± 2.40 129.1	−2.09 ± 3.09 128.2	0.16
Total cholesterol (mg/dL)	−2.7 ± 0.9 211.6	+3.01 ± 5.35 203.8	−0.853 ± 6.12 188.8	0.85

## Data Availability

De-identified patient data is available upon reasonable written request to the corresponding author.
